# Impact of Right Top Pulmonary Vein Location on Subcarinal Lymph Node Dissection in Thoracoscopic Esophagectomy: A Case Report and Literature Review

**DOI:** 10.70352/scrj.cr.24-0093

**Published:** 2025-02-28

**Authors:** Takeshi Horaguchi, Yuta Sato, Yuji Hatanaka, Yoshihiro Tanaka, Noriki Mitsui, Masahiro Fukada, Itaru Yasufuku, Ryuichi Asai, Jesse Yu Tajima, Nobuhisa Matsuhashi

**Affiliations:** Department of Gastroenterological Surgery and Pediatric Surgery, Gifu University Graduate School of Medicine, Gifu, Gifu, Japan

**Keywords:** esophageal cancer, right top pulmonary vein, pulmonary vein, subcarinal lymph nodes

## Abstract

**INTRODUCTION:**

The right top pulmonary vein (RTPV) is a rare anatomical variant that arises independently of the right superior lobe. It drains behind the right main bronchus or bronchus intermedius and into the left atrium or another pulmonary vein. This anomaly poses challenges during subcarinal lymph node dissection in thoracic surgery, such as esophagectomy, owing to the risk of vascular injury. The RTPV is mainly located behind the right main bronchus and right intermediate bronchus; however, reports of subcarinal dissection focusing on these sites are lacking. Herein, we present a case of esophageal cancer with an RTPV that was treated with thoracoscopic esophagectomy and propose a convenient classification for the anatomical findings and RTPV site.

**CASE PRESENTATION:**

A 71-year-old man underwent a thoracoscopic esophagectomy for esophageal cancer (T1bN0M0) during a routine medical checkup. A preoperative computed tomography scan revealed an anomaly in which the RTPV drained into the left atrium behind the right main bronchus. Radical subcarinal lymphadenectomy was performed while preserving the RTPV, using 3 dimensions for preoperative simulation and intraoperative navigation. The operation lasted 6 h and 42 min, and the blood loss volume was 30 mL. The patient’s postoperative course was uneventful, and he was discharged on postoperative day 21.

**CONCLUSIONS:**

In a retrospective review of esophageal cancer surgery cases at our hospital, RTPV was observed in 17/314 cases (5.4%). The most common inflow site was the inferior pulmonary vein (IPV) (9 cases), followed by the left atrium (5 cases), superior pulmonary vein (2 cases), and superior branch of the IPV (1 case). The inflow site was behind the right main bronchus and the right intermediate bronchus in 4 and 13 cases, respectively. Compared to past reviews, the inflow site varied somewhat; however, the vascular location remained the same. By classifying the areas behind the right main and right intermediate bronchi as Zones 1 and 2, respectively, cases in which the RTPV runs through Zone 1, as identified on preoperative computed tomography, should be manipulated with caution due to the risk of injury during lymph node dissection beneath the tracheal bifurcation.

## Abbreviations

3D
: 3-dimensional
CT
: computed tomography
IPV
: inferior pulmonary vein
LA
: left atrium
RTPV
: right top pulmonary vein
SCLNs
: subcarinal lymph nodes
SPV
: superior pulmonary vein
V6
: superior branch of IPV

## INTRODUCTION

There are many anatomical variations of the pulmonary veins, one of which is the right top pulmonary vein (RTPV). The RTPV is an anomalous blood vessel that runs from the right upper lobe into the left atrium (LA) or another branch of the pulmonary vein. Although these anomalies are rare, they can lead to damage and bleeding during the manipulation of subcarinal lymph node (SCLN) dissection in esophageal cancer surgery. Therefore, careful diagnosis via preoperative computed tomography (CT) is crucial to avoid critical intraoperative complications.

## CASE PRESENTATION

**Fig. 1:**
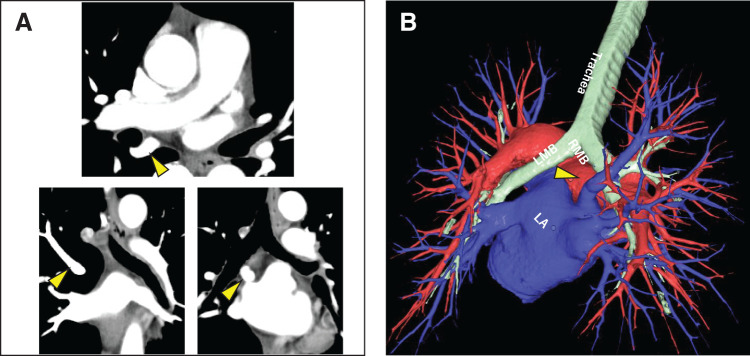
(**A**, **B**) Preoperative contrast-enhanced computed tomography and 3-dimensional (3D) imaging using Synapse Vincent (Fuji Photo Film Co., Ltd. ) showing the right top pulmonary vein behind the right main bronchus (yellow arrowhead). LMB, left main bronchus; RMB, right main bronchus; LA, left atrium

**Fig. 2:**
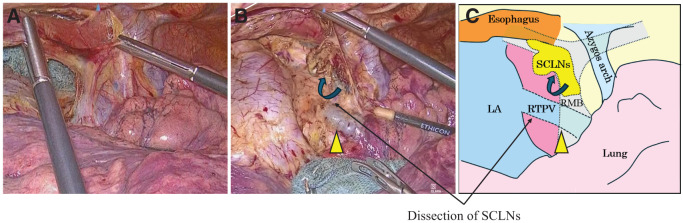
(**A, B**) Intraoperative findings and schematic illustration. During surgery, the right top pulmonary vein (yellow arrowhead) is preserved, and the subcarinal lymph nodes are dissected. (**C**) This figure shows a schematic diagram of (**B**). RMB, right main bronchus; LA, left atrium; SCLNs, subcarinal lymph nodes; RTPV, right top pulmonary vein

Our patient was a 71-year-old man. An endoscopic examination of the upper gastrointestinal tract revealed circumferential mucosal irregularities and small, elevated lesions in the middle thoracic esophagus. A histopathological diagnosis confirmed squamous cell carcinoma from the endoscopic biopsied specimens. Contrast-enhanced CT revealed no obvious lymph node metastasis, and the tumor was diagnosed as cT1b cN0 cM0 cStage I based on the Tumor Node Metastasis classification. In addition, 3-dimensional (3D) imaging using Synapse Vincent (FUJIFILM Co., Ltd., Tokyo, Japan) revealed a blood vessel flowing from the right upper lobe into the LA, leading to a preoperative diagnosis of RTPV (**Fig. 1**). Thoracoscopic esophagectomy was performed while the patient was in the left semi-lateral decubitus position. During surgery, the RTPV was identified behind the right main bronchus, as confirmed by preoperative CT imaging. The posterior pericardium was identified, and the RTPV was elevated by releasing the ventral fixation of the SCLNs to the dorsal free space. The RTPV was then carefully surrounded, and the SCLNs were mesenterically dissected, leaving only its fixation to the right main bronchus. The RTPV was preserved, and SCLN dissection was performed as usual (**Fig. 2**).^1)^ To avoid intraoperative injury of the RTPV caused by the digestive reconstruction procedure, the gastric tube was elevated via a retro-sternal route rather than a posterior mediastinal route and anastomosed to the cervical esophagus.^2)^ The operation lasted 6 h and 42 min; the blood loss volume was 30 mL. The postoperative course was uneventful, and the patient was discharged on postoperative day 21.

## DISCUSSION

An RTPV is an abnormal vessel among the pulmonary veins that was first reported by Webb et al. in a radiology department.^3)^ RTPV has also been reported by cardiologists and thoracic surgeons and is often mentioned by cardiologists in relation to venous catheter ablation for treating atrial fibrillation.^4)^ Anatomically, it arises independently of the right upper pulmonary vein and drains directly into the LA or another branch of the pulmonary vein behind the right bronchus. Advances in CT imaging technologies have enabled a more detailed anatomical understanding. Hagiwara et al. reported the utility of preoperative 3D-CT and highlighted its potential to reduce surgical time and postoperative complications.^5)^ At our institution, we create 3D-CT images using Synapse Vincent for all patients before esophageal cancer surgery, allowing for detailed anatomical assessments including vessel anomalies. Other anatomical anomalies have been observed beyond this particular instance.

In a retrospective study of cases of esophageal cancer at our hospital from January 2019 to July 2024, RTPV was observed in 17 of 314 cases (5.4%; **Table 1**). The median RTPV diameter was 5.5 mm. The breakdown of the inflow site was as follows: inferior pulmonary vein (IPV) in 9 cases (53%), LA in 5 cases (29%), superior pulmonary vein (SPV) in 2 cases (12%), and superior branch of the IPV (V6) in 1 case (6%). The RTPV primarily runs behind the right bronchus, but we found only 4 cases (24%) where it ran behind the right main bronchus. In most cases (13 cases, 76%), it ran behind the right intermediate bronchus. All the RTPVs running behind the right main bronchus were visible during surgery. In contrast, only some of the RTPVs that ran behind the right middle bronchus were visible during surgery.

**Table 1:** Patients with esophageal cancer and a right top pulmonary vein at our hospital

No	Age	Sex	Diameter (mm)	Inflow-site	Location	Visual recognition
1	64	M	5.42	LA	BMB, BIB	Yes
2	62	M	5.8	LA	BMB	Yes
3	56	M	3.36	LA	BIB	No
4	76	M	6.2	IPV	BIB	No
5	65	M	4.6	IPV	BIB	Yes
6	72	M	3.43	IPV	BIB	No
7	65	M	5.07	LA	BMB	Yes
8	75	M	4.53	IPV	BIB	No
9	74	M	6.8	IPV	BIB	No
10	70	M	5.54	IPV	BIB	No
11	52	M	2.27	V6	BIB	No
12	69	M	10.29	SPV	BMB	Yes
13	72	M	5.1	IPV	BIB	No
14	80	F	3.1	IPV	BIB	No
15	76	M	4.33	IPV	BIB	No
16	81	M	6.4	SPV	BIB	No
17	71	M	11	LA	BMB	Yes

LA: left atrium; IPV: inferior pulmonary vein; V6: superior branch of IPV; SPV: superior pulmonary vein; BMB: behind the right main bronchus; BIB: behind the right intermediate bronchus.

**Fig. 3:**
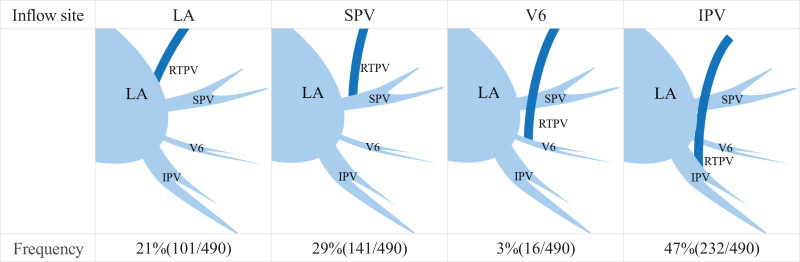
Frequency of RTPV inflow sites based on literature reviews. LA, left atrium; IPV, inferior pulmonary vein; V6, superior branch of IPV; SPV, superior pulmonary vein; RTPV, right top pulmonary vein

**Table 2** summarizes the previous reviews of RTPVs.^4,6–15)^ Overall (including our study), the frequency of RTPV was 4.46%, and the median RTPV diameter was 5.8 mm. The most common inflow site was the IPV (47.35%) (**Fig. 3**). The frequencies of each running pattern behind the right main bronchus and behind the right intermediate bronchus were 4.83% and 95.17%, respectively.

**Table 2:** Literature review of the right top pulmonary vein

Author	Year	RTPV *n* (%)	Vascular diameter	Inflow site				Location	
				IPV	LA	SPV	V6	BMB	BIB
Web et al.^3)^	1984	1/40 (2.5)	–	0	1	0	0	0	1
Jardin^19)^	1986	10/107 (9.3)	–	10	0	0	0	0	10
Kim^20)^	1995	14/280 (5)	–	11	0	3	0	0	14
Matsubara et al.^6)^	2003	2/700 (0.28)	–	–	–	–	–	0	1
Kato^21)^	2003	2/55 (3.63)	–	0	2	0	0	–	–
Lickfett et al.^7)^	2004	3/91 (3.29)	7	0	3	0	0	1	–
Asai^22)^	2005	41/725 (5.7)	4.1	17	0	22	2	0	41
Weerasooriya et al.^4)^	2005	1/84 (1.19)	13	0	1	0	0	–	–
Kaseno^23)^	2008	16/428 (3.73)	5.6	0	16	0	0	–	–
Arslan et al.^8)^	2008	14/610 (2.29)	5.1	–	–	–	–	0	14
Yamada et al.^9)^	2010	1/86 (1.16)	–	0	1	0	0	0	1
Akiba^24)^	2010	3/140 (2.14)	–	0	3	0	0	0	3
Akiba et al.^10)^	2013	10/303 (3.3)	–	0	4	4	1	2	9
Shi et al.^11)^	2017	1/102 (0.98)	–	0	1	0	0	–	–
Miyamoto et al.^12)^	2021	31/387 (8.01)	2.2	13	11	3	4	0	31
Yaginuma et al.^13)^	2021	154/4673 (3.29)	3.7	65	3	85	0	–	–
Murota et al.^14)^	2023	131/1437 (9.1)	–	85	29	16	–	–	–
Li et al.^15)^	2023	57/600 (9.5)	–	22	21	6	8	–	–
Our study (Horaguchi et al.)	2024	17/314(5.4)	5.1	9	5	2	1	4	13

RTPV: right top pulmonary vein; LA: left atrium; IPV: inferior pulmonary vein; V6: superior branch of IPV; SPV: superior pulmonary vein; BMB: behind the right main bronchus; BIB: behind the right intermediate bronchus

**Fig. 4:**
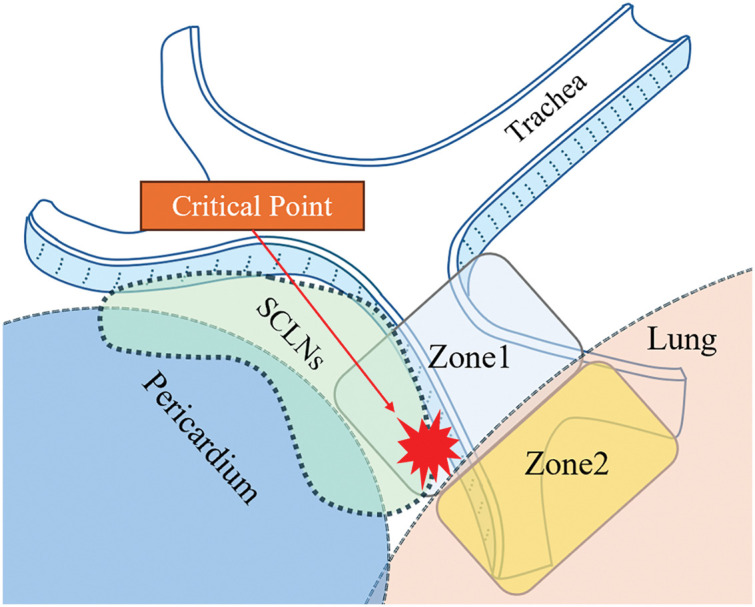
Classification of the right top pulmonary vein location. Zone 1 is defined as the area behind the right main bronchus, and Zone 2 is defined as the area behind the right intermediate bronchus. Critical points are areas that require special attention. SCLNs, subcarinal lymph nodes

**Fig. 5:**
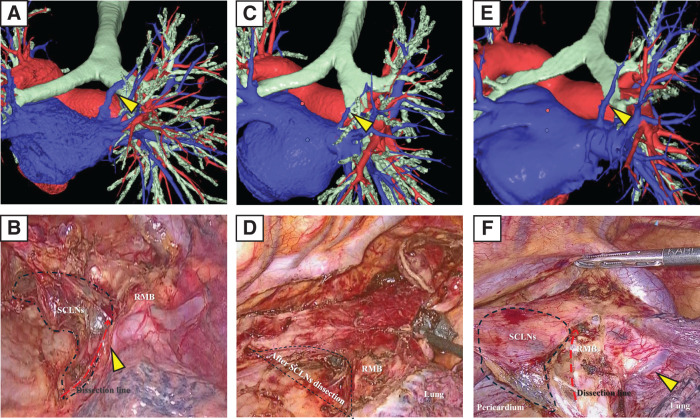
Representative cases. (**A**, **B**) The right top pulmonary vein (yellow arrowhead) runs through Zone 1 and through the subcarinal dissection area. (**C**, **D**) The right top pulmonary vein runs through Zone 2, but it is not confirmed in the surgical field. (**E**, **F**) The right top pulmonary vein (yellow arrowhead) runs through Zone 2. It is confirmed in the surgical field but does not contribute to the dissection of subcarinal lymph nodes. SCLNs, subcarinal lymph nodes; RMB, right main bronchus

Xu et al.^16)^ reported that the metastasis rates to SCLNs for upper, middle, and lower thoracic esophageal cancer and oesophagogastric junction cancer were 7.14%, 38.00%, 1.45%, and 10.00%, respectively. These findings support the great oncological benefit of thorough SCLN dissection. In addition, the guidelines define SCLN as regional lymph nodes in thoracic esophageal cancer and esophagogastric junction cancer.^17)^ Therefore, a proper understanding of the anatomy around the carina is essential, and a common anatomical guide for RTPV is needed. Akiba et al.^10)^ proposed classifying RTPVs into 6 types based on their locations and inflow site. However, in this study, we divided the zones into 2 for convenience, focusing on the vein’s location and the risk of damage during dissection (**Fig. 4**). The area behind the right main bronchus is classified as Zone 1 and the area behind the right intermediate bronchus is classified as Zone 2. In cases where the RTPV runs through Zone 1, careful surgical manipulation is required as the RTPV may be damaged during SCLN dissection; thus, identifying a Zone 1 RTPV on preoperative CT images is crucial (**Figs. 5A**, **5B**). RTPV running through Zone 2 may not be visible in the surgical field. Even if it is confirmed, it may not affect the difficulty of the SCLN dissection procedure (**Figs. 5C**–**5F**). In particular, visually confirming the RTPV in the Zone 1 area near Zone 2 is difficult because of the presence of the pulmonary parenchyma, and there is a risk of traumatic damage when cutting the mediastinal pleura; therefore, careful manipulation is required (critical point in **Fig. 4**). Because the RTPV runs inside the subcarinal lymph nodes, even in laparoscopic or robot-assisted surgery, which offers the advantage of magnified vision, there remains a risk of injury, so sufficient caution is required.

Intraoperative injury of the RTPV can cause massive bleeding and cardiac tamponade due to pericardial bleeding.^7,8)^ Sato et al. reported that the key to preserving the RTPV and safely performing SCLN dissection is performing surgery while being aware of the layers and membranes.^1)^ In the present case, the diameter of the RTPV was 11 mm, but the SCLNs could be safely dissected by paying attention to the layer structure and the presence of the RTPV. Additionally, when considering vascular diameter, RTPVs larger than 4.5 mm require caution to prevent injury, and previous reports have indicated that ligation should be avoided to prevent pulmonary congestion.^3)^ According to Mikami et al.,^18)^ the maximum RTPV diameter in previous reports was 7.8 mm; thus, our case had the thickest RTPV diameter among previously reported cases. The size of this vessel suggests that it plays an important role as a pivotal drainage vein for the right upper lobe of the lung, and preserving it contributes greatly to the postoperative course.

## CONCLUSIONS

This is the first report to classify the RTPV location and outline precautions during subcarinal dissection. Furthermore, the patient in this case had the thickest RTPV diameter reported to date. We expect that this anatomical classification could help guide preoperative imaging diagnoses and thoracoscope- or robot-assisted surgery for esophageal cancer to avoid critical intraoperative complications.

## ACKNOWLEDGMENTS

We would like to thank Editage (www.editage.jp) for editing the English language.

## DECLARATIONS

### Funding

There is no funding to report.

### Authors’ contributions

TH and YS designed the case report. YH, YT, and NMi performed the experiments.

MF, IY, RA, and JYT collected the data. NMa confirmed the authenticity of the raw data.

All the authors have read and approved the final version of the manuscript.

### Availability of data and materials

The datasets used and/or analyzed in the current study are available from the corresponding author upon reasonable request.

### Ethics approval and consent to participate

The present study was conducted according to the principles of the Declaration of Helsinki and was approved by the Ethical Review Board of the Gifu University Hospital (approval no. 2024-221; November 11, 2024). The opt-out method was applied to obtain consent for participation in the present study using a home page.

### Consent for publication

Written informed consent was obtained from the patient’s guardians for the publication of the accompanying images or data included in this article.

### Competing interests

The authors declare that they have no competing interests.
